# Identification and Characterization of Shaker K^+^ Channel Gene Family in Foxtail Millet (*Setaria italica*) and Their Role in Stress Response

**DOI:** 10.3389/fpls.2022.907635

**Published:** 2022-06-09

**Authors:** Ben Zhang, Yue Guo, Hui Wang, Xiaoxia Wang, Mengtao Lv, Pu Yang, Lizhen Zhang

**Affiliations:** ^1^State Key Laboratory of Sustainable Dryland Agriculture, Shanxi Agricultural University, Taiyuan, China; ^2^School of Life Sciences, Shanxi University, Taiyuan, China

**Keywords:** shaker K^+^ channel, foxtail millet (*Setaria italica*), gene regulation, abiotic stress, phytohormone

## Abstract

Potassium (K^+^) is one of the indispensable elements in plant growth and development. The Shaker K^+^ channel protein family is involved in plant K^+^ uptake and distribution. Foxtail millet (*Setaria italica*), as an important crop, has strong tolerance and adaptability to abiotic stresses. However, no systematic study focused on the Shaker K^+^ channel family in foxtail millet. Here, ten Shaker K^+^ channel genes in foxtail millet were identified and divided into five groups through phylogenetic analysis. Gene structures, chromosome locations, cis-acting regulatory elements in promoter, and post-translation modification sites of Shaker K^+^ channels were analyzed. *In silico* analysis of transcript level demonstrated that the expression of Shaker K^+^ channel genes was tissue or developmental stage specific. The transcription levels of Shaker K^+^ channel genes in foxtail millet under different abiotic stresses (cold, heat, NaCl, and PEG) and phytohormones (6-BA, BR, MJ, IAA, NAA, GA3, SA, and ABA) treatments at 0, 12, and 24 h were detected by qRT-PCR. The results showed that *SiAKT1*, *SiKAT3, SiGORK*, and *SiSKOR* were worth further research due to their significant responses after most treatments. The yeast complementation assay verified the inward K^+^ transport activities of detectable Shaker K^+^ channels. Finally, we found interactions between SiKAT2 and SiSNARE proteins. Compared to research in Arabidopsis, our results showed a difference in SYP121 related Shaker K^+^ channel regulation mechanism in foxtail millet. Our results indicate that Shaker K^+^ channels play important roles in foxtail millet and provide theoretical support for further exploring the K^+^ absorption mechanism of foxtail millet under abiotic stress.

## Introduction

As the main abiotic stresses, drought, salt, and temperature stresses severely affect plant growth and development. Potassium (K^+^) is one of the essential nutrients for plants and is involved in many plant physiological processes, including plant cell turgor maintenance, stomata movement, photosynthesis, carbon assimilation, and osmotic balance (Wang et al., 2013). K^+^ is also recognized as a key limiting factor in agricultural production. Moreover, many reports have suggested the protective role of K^+^ under abiotic stress in different species (Gong et al., 2020; Ma et al., 2020).

In plants, there are two main mechanisms for K^+^ absorption, the high-affinity K^+^ absorption mechanism mediated by transporters and the low-affinity K^+^ absorption mechanism mediated by K^+^ channels (Raddatz et al., 2020). The K^+^ channels are divided into three families, including Shaker, TPK, and Kir-like K^+^ channel families (Lebaudy et al., 2007). Among them, the Shaker K^+^ channel is the most extensively studied gene family and has been identified in many plant species, including Arabidopsis (Dreyer and Uozumi, 2011), rice (Amrutha et al., 2007; Hwang et al., 2013), maize (Bauer et al., 2000), poplar (Zhang et al., 2010), sweet potato (Jin et al., 2021), and soybean (Feng et al., 2021). Shaker K^+^ channels are involved in K^+^ uptake from soil and transport among different tissues. Their subunits share similar conserved structural features. All of them contain six transmembrane α-helices called S1–S6. They form a functional channel as homo- or hetero-tetrameric assemblies, which mediates the transport of K^+^ across the membrane (Dreyer and Blatt, 2009). In Arabidopsis, the nine Shaker K^+^ channel proteins can be divided into five groups (Pilot et al., 2003b). Group I (*KATs*) and Group II (*AKT1, AKT5*, and *AKT6*) are inward rectification channels whose opening are regulated by the negative membrane voltage. Group V (*SKOR* and *GORK*) is the outward rectification channel whose opening requires positive membrane voltages. Group III (*AKT2*) belongs to the weak inward rectification channel, and Group IV contains a silent subunit *KC1* (Lebaudy et al., 2007).

The role of the Shaker K^+^ channel in plant abiotic stress responses has been well studied in many plant species. In Arabidopsis, most Shaker K^+^ channels are involved in the opening and closing of stomata under drought stress (Hosy et al., 2003; Lebaudy et al., 2008). The cold and salt stress-induced expression of *GORK* leads to disruption of cytosolic Na^+^/K^+^ ratio and suppression of plant K^+^-dependent metabolism (Becker et al., 2003; Chen et al., 2007). Over-expressing *OsAKT1* in rice elevates the tissue K^+^ content and improves plant drought tolerance (Ahmad et al., 2016). A similar phenomenon has been reported for *HvAKT1* over-expression in barley (Feng et al., 2020). ZMK1, a homolog of AKT1 in *Zea May*, mediates K^+^ uptake in coleoptile cells and its expression is induced by auxin (Philippar et al., 1999, 2006). Over-expressing *GmAKT1* of soybean in Arabidopsis enhances root length and K^+^ concentration under drought and salt stress, suggesting that *GmAKT1* plays a role in soybean response to such stresses (Feng et al., 2021). The GORK channel associated K^+^ efflux and the channel’s sub-cellular location in the guard cell is regulated by ABA, a drought-related phytohormone, through the OPEN STOMATA 1 (OST1) kinase involved signaling system (Chen et al., 2021). Ooi et al. have found that direct GORK-ABA interaction increases the K^+^ efflux current (Ooi et al., 2017). The inward rectification channel KAT1 is also regulated by ABA (Sutter et al., 2007). Recent reports suggest that KAT1 interacts with the membrane traffic related SNARE family protein SYP121, which regulates the ABA related channel trafficking and directly induces the channel activity through protein-protein interaction (Sutter et al., 2006; Honsbein et al., 2009). Further research reports that another SNARE protein, VAMP721, the partner of SYP121 during membrane fusion, also binds with K^+^ channels but regulates the channel activity in an opposite way (Zhang et al., 2015). The expression of SNARE protein is up-regulated by abiotic stresses (Kwon et al., 2020; Wang et al., 2021). Thus, the SNARE related regulation of K^+^ channels might be the key to understanding the mechanism of K^+^-dependent plant abiotic tolerance.

*Setaria italica* is a Poaceae crop with strong environmental adaptability. The high abiotic stress tolerance of foxtail millet leads to its broad cultivation area in Asia and Africa (He et al., 2015; Peng and Zhang, 2020). In 2012, the foxtail millet genome was sequenced, making it possible to identify genes and study their functions (Bennetzen et al., 2012; Zhang et al., 2012; Tan et al., 2022). Furthermore, its relatively small genome makes foxtail millet a promising C4 model plant (He et al., 2015). Previous research from our group has analyzed the role of SNARE proteins under drought stress in foxtail millet (Wang et al., 2021). The high-affinity K^+^ transporters in foxtail millet have also been analyzed (Zhang et al., 2018). However, little research has focused on the Shaker K^+^ channels in foxtail millet and their role in stress response. Therefore, we identified the Shaker K^+^ channel genes from the foxtail millet database and obtained ten members. These genes were further analyzed *via* phylogeny, conserved motifs and domain, promoter, and tissue expression patterns. Then, quantitative real-time PCR (qRT-PCR) was used to analyze the transcription levels of Shaker K^+^ channel genes in foxtail millet under different abiotic stresses and phytohormone treatments. The K^+^ transport activities of these genes were verified by yeast complementation assay. In addition, we also investigated the interaction between Shaker K^+^ channel proteins and SNARE proteins in foxtail millet. These analyses explored the mechanism of Shaker K^+^ channel-related K^+^ absorption in foxtail millet and provided directions for future research.

## Materials and Methods

### Identification of Shaker K^+^ Channel Genes in Foxtail Millet

The Shaker K^+^ channel gene sequences of Arabidopsis and rice were extracted from the Arabidopsis Information Resource^1^ and the Rice Genome Annotation Project.^2^ These sequences were blasted in the Phytozome database (Phytozome 13, *Setaria italica* v2.2) (Goodstein et al., 2012) to obtain the candidate shaker K^+^ channel coding sequences. Then the Arabidopsis channel sequence was submitted to the Pfam database^3^ to obtain the hidden Markov model (HMM) profile of the Shaker K^+^ channel conserved domain, which were queried against the candidate sequences of Shaker K^+^ channels from foxtail millet. The protein sequences of these candidate genes were further submitted to the SMART program.^4^ Finally, the sequences with a complete channel conserved domain were selected for subsequent analysis. Based on the protein sequence, the theoretical isoelectric point (PI), molecular weight (MW), and amino acid composition of the Shaker K^+^ channels from foxtail millet were analyzed by ExPASY^5^ (Gasteiger et al., 2003). Their subcellular localization was predicted by WoLF PSORT^6^ (Horton et al., 2007). The protein transmembrane region of the Shaker K^+^ channel was predicted *via* the TMHMM 2.0^7^ (Krogh et al., 2001).

### Phylogenetic Analysis and Chromosome Location of Shaker K^+^ Channel Family

The amino acid sequences of Shaker K^+^ channels from foxtail millet, Arabidopsis (Cao et al., 1995), and rice (Hwang et al., 2013) were homologously aligned by the ClustalX software (Thompson et al., 2003) with default parameters. Based on this comparison, MEGA7.0 (Kumar et al., 2016) software was used to construct the phylogenetic tree of the Shaker K^+^ channels with the following parameters: Poisson model and pairwise deletion, Bootstrap 1,000 repetitions. The gene annotations of candidate Shaker K^+^ channel genes were extracted from the Phytozome database (Phytozome 13, *Setaria italica* v2.2) (Goodstein et al., 2012), from which the position of the genes on the chromosome was obtained, and the physical map was drawn using the MapChart software (Voorrips, 2002).

### Motif Composition and Gene Structure Analysis of Foxtail Millet Shaker K^+^ Channel Genes

The conserved protein motifs of Shaker K^+^ channels from foxtail millet were predicted by the online Multiple Em for Motif Elicitation (MEME) program^8^ (Bailey et al., 2009), with the following parameters: the number of repetition = any, the maximum number of motifs = 10. These genes’ CDS and genome sequences were downloaded from the Phytozome database and submitted to Gene Structure Display Server online program (GSDS^9^) (Hu et al., 2015) for gene structure analysis.

### Cis-Acting Element Analyses

The 2,000 bp upstream sequences of Shaker K^+^ channel gene in foxtail millet were extracted from the Phytozome database and submitted to the PlantCARE to obtain the cis-elements in the promoter regions^10^ (Lescot et al., 2002).

### Tissue Expression Patterns of Shaker K^+^ Channel in Foxtail Millet

To study the potential expression patterns of Shaker K^+^ channels from foxtail millet at different tissues and developmental stages, the fragments per kilobase of the exon model per million mapped (FPKM) values of these genes were obtained from the GeneAtlas v1 Tissue Sample (Phytozome 13), including the data of etiolated seeding (5 days), germ shoot (6 days), shoot (1 week), leaf [different leaf (1, 2, 3, 4, 5, 6) at 2 weeks], panicle stage 1 and 2, and root (10 days). These data were submitted to TBtools (Chen et al., 2020) for expression profile mapping.

### Plant Growth Conditions and Treatment

The foxtail millet cultivars “Jingu21,” “Longgu16,” and “Jigu39” were used in the present study. Seeds are obtained from Prof. Lizhen Zhang’s lab (School of Life science, Shanxi University, China). The plant seeds were grown in a tray containing vermiculite and nutrient soil at a ratio of 1:1 and cultivated under greenhouse conditions (16 h light/8 h dark at 23–26°C, 50,000 Lux light, and 30–50% relative humidity). When the seeds were germinated and grown to the two-leaf stage (4 days), select seedlings with consistent growth stages were transferred to plastic pots with three plants per pot. For temperature stress, after another 14 days, “Jingu21” seedlings were separately subjected to heat (40°C day/32°C night) and cold (4°C) stress in a constant temperature incubator for 0, 12, and 24 h. For other abiotic stresses, 10 days old “Jingu21” seedlings were transferred to 1/2 MS liquid medium and cultured for another 4 days, then subjected to salt (150 mmol/l, 200 mmol/l NaCl), PEG6000 (10%, 15%), ABA (100 μmol/l), 6-BA (75 μmol/l), IAA (10 μmol/l), NAA (10 nmol/l), BR (100 μmol/l), GA3 (1 mmol/l), MJ (100 μmol/l), and SA (10 mmol/l) treatments for 0, 12, and 24 h. There were three replicates for each stress treatment. The leaves were harvested. In addition, the leaves and roots of “Jingu21,” “Longgu16,” and “Jigu39” seedlings grown for 14 days under normal conditions were also collected. All samples were immediately frozen in liquid nitrogen and stored at −80°C for further RNA extraction.

### RNA Extraction and qRT-PCR

Total RNA was extracted from samples using TransZol™ UP Plus RNA Kit (TransGen Biotech, Beijing, China), according to the manufacturer’s instructions. The first-strand cDNA templates were synthesized using EasyScript ^®^ One-Step gDNA Removal and cDNA Synthesis SuperMix Kit (TransGen Biotech, Beijing, China) according to the manufacturer’s instructions. The total volume of the qRT-PCR reaction system was 10 μl, which included 5 μl of TransStart ^®^ Tip Green qPCR SuperMix (TransGen Biotech, Beijing, China), 1 μl of diluted cDNA template, 0.8 μl of upstream and downstream primers (10 μmol/l each), and 3.2 μl of RNase free ddH_2_O. The qRT-PCR thermal cycler program included 94°C for 30 s, followed by 45 cycles at 94°C for 15 s and 58°C for 30 s. All primers were synthesized by Shanghai Sangon Biotech (As shown in Supplementary Table 1), wherein *SiAct2 (Seita.8G043100)* was used as the internal reference. Each experiment included three technical replicates and three biological replicates.

### Prediction of Potential *N*-Glycosylation and Phosphorylation Sites of Shaker K^+^ Channels

The Shaker K^+^ channel protein sequences were submitted to NetOGlyc 4.0^11^ for *N*-glycosylation site prediction. The NetPhos 3.1^12^ was used to predict protein phosphorylation sites (including Serine, Threonine, and Tyrosine). The positive result was confirmed when the score was higher than 0.5.

### Abscisic Acid and Guanine Nucleotide-Binding Proteins Binding Site Prediction in Shaker K^+^ Channels

The presence of abscisic acid (ABA) interaction domain (D543–G575) (Ooi et al., 2017) and guanine nucleotide-binding proteins (G-protein) binding motif (E165–Y169) (Deupi et al., 2012; Adem et al., 2020) in the protein sequence of Arabidopsis AtGORK (At5G37500) have been reported. Due to the high sequence similarity between GORK and SKOR, it is worth predicting whether similar binding sites exist in other plants, especially foxtail millet. The GORK and SKOR amino-acid sequences of Arabidopsis (GORK, At5G37500; SKOR, At3G02850), tobacco (GORK, XP_016457756; SKOR, XP_016460239), foxtail millet (GORK, Seita.7G111600; SKOR, Seita.4G110300), rice (GORK, Os04g36740; SKOR, Os06g14030), sorghum (GORK, Sobic.006G093400; SKOR, Sobic.010G102800), and wheat (GORK, Traes_2BL_C71ACBCED; SKOR, Traes_7DS_965990A2F) were extracted from the Phytozome and NCBI database, and multiple sequence alignments were performed using the ClustalX software.

### Molecular Cloning and Vector Construction

Open reading frames for *SiVAMP721 (Seita.6G010600)*, *SiSNAP33 (Seita.9G493300)*, *SiSYP121 (Seita.9G063400)*, and Shaker K^+^ channel genes were amplified from “Jingu21” with gene-specific primers including Gateway attachment sites (attB1/attB2; as shown in Supplementary Table 1). These amplified products were inserted into the pDONR207 vector to obtain entry clones by BP reactions using BP Clonase II (Invitrogen) and verified *via* sequencing (Sangon Biotech, Shanghai, China).

For the mating-based Split-Ubiquitin System (mbSUS) assay, destination clones for Shaker K^+^ channel genes were used as bait and recombined in the pMetYC-Dest vector. *SiSNAREs* as prey were recombined in the pNX35-Dest vector (Grefen et al., 2009). These clones were generated using LR Clonase II (Invitrogen) by LR reaction according to the manufacturer’s instructions.

For yeast complementation assay, destination clones for all Shaker K^+^ channels were recombined in padh1-Dest vector by LR reaction. The padh1-Dest vector was created by replacing TRP1 in pZMT-Dest (Grefen, 2014) with LEU1. The sequence containing LEU1 with a NdeI site at the 3′ and a NruI site at the 5′ termini was produced by DNA synthesis (Sangon Biotech, Shanghai, China) and digested by NdeI and NruI (NEB). The backbone from pZMT-Dest was amplified using primers BZ-S-NruI-ZMT and BZ-A-NdeI-ZMT. T4 DNA ligase (NEB) was used to carry out ligation. The subsequent transformation and selection were in ccdB-survival cells (TransGen Biotech, Beijing, China). The vector sequence was verified by restriction endonuclease digestion and sequencing. As shown in Figure 6A, the core expression area of padh1-Dest contains a Gateway cassette (attR1/attR2) with a C-terminal Myc tag under the control of the *adh1* promoter.

### Yeast Complementation Assay

The full-length coding sequences of Shaker K^+^ channels in foxtail millet were inserted into yeast expression vector padh1-Dest and transformed into the yeast mutant strain R5421 (MATα ura3-52 leu2 trk1Δ his3Δ200 his4-15 trk2Δ1::pCK64; Weidi Biotech, Shanghai, China), in which two K^+^ transporters, TRK1 and TRK2, were deleted (Li et al., 2014). Wild-type yeast strain R757 was used as the positive control. The yeast complementation experiment was performed as described previously (Li et al., 2014; Han et al., 2016). A commercial Myc antibody (Abcam) was used in immunoblot analysis to verify protein expression.

### Mating-Based Split-Ubiquitin Assays

The mbSUS assays were performed as described before (Grefen et al., 2009; Horaruang and Zhang, 2017). Bait destination clones were transformed into yeast strain THY.AP4 and prey clones were transformed into THY.AP5. Pools of 10–15 yeast colonies were selected and inoculated in selective media (CSM_–LM_ for bait in THY.AP4 and CSM_–MTU_ for prey in THY.AP5) and grown at 28°C, 180 rpm overnight. The liquid cultures were harvested and resuspended in the yeast peptone dextrose (YPD) medium for yeast mating. Equal aliquots of yeast containing bait and prey were mixed in sterile PCR tubes, dropped on the YPD plate, and cultured at 28°C overnight. The colonies were transferred from the YPD plate onto the CSM_–LMTU_ plate and incubated at 28°C for 2–3 days. Diploid colonies were selected and inoculated in liquid CSM_–LMTU_ medium and grown at 180 rpm and 28°C overnight. After that, yeast was harvested by centrifugation and resuspended in sterile water. Serial dilutions at OD600 = 1 and 0.1 in water were dropped (5 μl per spot) on CSM_–AHLMTU_ plates with added Met. Plates were incubated at 28°C, and images were taken after 3 days. Yeast was also dropped on CSM_–LMTU_ control plates to confirm mating efficiency and cell density, and growth was imaged after 24 h incubation at 28°C. To verify the expression of bait and prey, yeast was harvested and extracted for protein immunoblot analysis using commercial HA antibody (Abcam) for prey and commercial VP16 antibody (Abcam) for bait.

### Statistical Analysis

In the present study, the statistical analysis was reported as means ± SE with significance determined by Student’s *t*-test or ANOVA in the SigmaPlot v.11.2 (Systat Software).

## Results

### Identification of Shaker K^+^ Channel Genes in Foxtail Millet

The coding sequences of Shaker K^+^ channels from Arabidopsis and rice were used to blast in the Phytozome foxtail millet database, and 12 candidate genes were obtained. Then, the Arabidopsis Shaker K^+^ channel protein sequences were submitted to the Pfam database (see footnote 3) to recognize the channel domains (PF00520.31, PF00027.29, and PF11834.8). The protein sequences of these candidate genes were submitted to the SMART program, and 10 genes were identified as Shaker K^+^ channels in foxtail millet (Table 1). The foxtail millet Shaker K^+^ channel proteins were 505–903 amino acids in length, and molecular weight was distributed from 58.16 to 98.76 kDa. The isoelectric point of Shaker K^+^ channel proteins ranged from 6.23 to 9.08. Subcellular localization predictions indicated that all Shaker K^+^ channel proteins were localized on the plasma membrane with 4–6 trans-membrane segments (Table 1).

**TABLE 1:** Detailed information of predicted Shaker K^+^ channel genes in foxtail millet.

Gene ID	Genome position		Chr	Length (aa)	pI	Mw	Predicted name	Subcellular localization	Number of TM segments	Orthologous gene ID
					
	Start	End				(KDa)				
Seita.1G020100	1728964	1735893	chr1	761	7.6	87.45	SiKAT3	PM	5	AT2G25600, AT4G18290, AT4G32500, AT5G46240, Os02g14840
Seita.5G244400	3068192	30688913	chr5	885	6.66	99.66	SiAKT1	PM	5	AT2G26650, Os01g45990
Seita.4G110300	103449	10351307	chr4	854	6.23	96.77	SiSKOR	PM	5	AT3G02850, Os06g14030, AT5G37500
Seita.3G233000	19292761	19297970	chr3	855	8.58	94.53	SiAKT2/3	PM	5	AT4G22200, Os05g35410
Seita.5G298000	35360171	35362824	chr5	617	8.92	69.5	SiKC1a	PM	6	AT4G32650, Os06g14310, Os04g02720, Os01g52070
Seita.7G111600	21144981	21149199	chr7	724	8.47	81.79	SiGORK	PM	5	Os04g36740
Seita.5G323200	37290994	37293812	chr5	505	8.95	58.16	SiKAT1	PM	5	Os01g55200
Seita.5G148000	1317289	13177173	chr5	695	8.82	79.48	SiKAT2	PM	4	Os01g11250
Seita.2G049100	3916917	3920473	chr2	903	8.32	98.76	SiAKT2	PM	5	Os07g07910
Seita.1G210600	28923321	28925051	chr1	576	9.08	63.15	SiKC1b	PM	4	Os06g14310

### Phylogeny Analysis of Shaker K^+^ Channel Genes in Foxtail Millet

**FIGURE 1:**
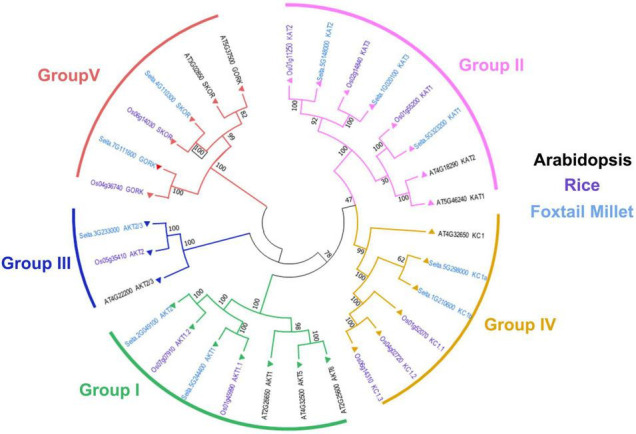
Phylogenetic analysis and classification of Shaker K^+^ channel proteins from foxtail millet (light blue), Arabidopsis (black), and rice (purple). Shaker K^+^ channels are divided into five distinct groups denoted by different colored lines.

To further explore the evolutionary relationship of Shaker K^+^ channels among different species, the protein sequences from foxtail millet, Arabidopsis, and rice were used to construct a phylogenetic tree. As shown in Figure 1, the Shaker K^+^ channel genes from foxtail millet can be classified into five groups based on their phylogenetic relationships and functional divergences (Pilot et al., 2003b; Hwang et al., 2013). Group I and II were inward rectifying channels. There were *SiAKT1, SiAKT2* in Group I and *SiKAT1, SiKAT2*, and *SiKAT3* in Group II. Only *SiAKT2/3* belongs to Group III. Group IV, the regulatory or silent channel, included *SiKC1a* and *SiKC1b*. Group V was the outward rectifying channel, including *SiGORK* and *SiSKOR*. Similar groups of Shaker K^+^ channels from different species suggested that this protein family was evolutionary conserved. Compared to Arabidopsis, proteins from foxtail millet were more similar to their orthologous in rice.

### Chromosome Location, Gene Structure, and Motif Analyses of Shaker K^+^ Channel Gene Family

Shaker K^+^ channel genes were distributed on 6 out of 9 chromosomes of foxtail millet (Supplementary Figure 1). Chromosome 1 contained 2 channel genes (*SiKAT3* and *SiKC1b*). Only one gene on chromosome 2, 3, 4, and 7. Chromosome 5 contained the greatest number of Shaker K^+^ channel genes (4 genes).

**FIGURE 2:**
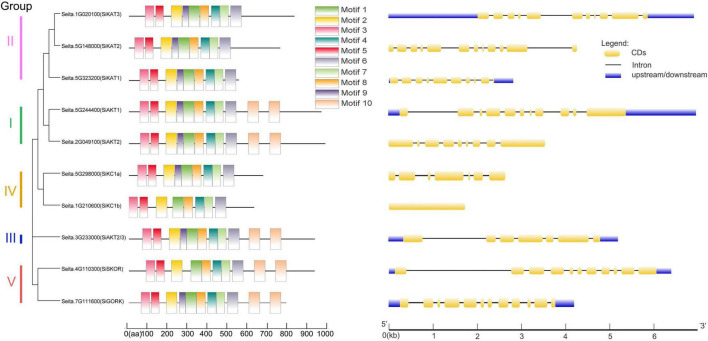
Protein motifs and gene structures of Shaker K^+^ channels from foxtail millet. **(Left)** In the Shaker K^+^ channel proteins, 10 motifs were identified by the MEME tool, represented by different colors (1–10) and depicted by TBtools. **(Right)** The exon-intron structure of these Shaker K^+^ channel genes was predicted by GSDS 2.0. The yellow boxes represent the coding sequence (CDs), the black lines represent introns, and the blue boxes represent up/downstream untranslated regions (UTR).

Next, the gene structural diversity and protein motif distribution of Shaker K^+^ channels in foxtail millet were explored. As shown in Figure 2 at left, ten conserved motifs were detected in Shaker K^+^ channel proteins by MEME. All channel proteins contained conserved motifs 1–8. Proteins in Group I, III, and V contained two copies of motifs 10. Motif 9 were missing in *SiSKOR* and *SiKC1b*. Channels in the same group containing similar conserved motifs also supported our classification based on the phylogenetic tree.

As shown on the right side of Figure 2, the same group of genes contained similar numbers of introns and exons. For example, Group I, II, and V contained 8–11 introns, and group III and IV contained 6 introns except for *SiKC1b*, which contained no intron. The differences in gene structures indicated the evolutionary and functional differences in the Shaker K^+^ channel family and provided additional evidence to support phylogenetic grouping.

### Cis-Acting Regulatory Elements Analysis of Shaker K^+^ Channel Genes

To further understand the regulatory mechanism of the Shaker K^+^ channel genes, the 2,000 bp promoter regions upstream of them were used for cis-elements analysis. As shown in Supplementary Table 2, 68 elements were identified. Based on the functional differences, important cis-acting elements were divided into three major groups: plant growth and development related, phytohormone responsiveness related, and abiotic/biotic stresses related cis-acting elements. As shown in Supplementary Figure 2, circadian element (Anderson et al., 1994) was only found in *SiKC1a*, indicating that this gene may be related to photoperiod regulation. The RY element is a seed-specific promoter which mediates initial transcriptional activation during embryo mid maturation (Lelievre et al., 1992; Guerriero et al., 2009). This cis-acting element was only predicted in *SiKAT3*. In addition, promoter elements related to plant growth, such as A-box, CAT-box, and O2-site, were found in 3, 6, and 6 Shaker K^+^ channel genes, respectively. Abscisic acid (ABA) response elements (ABREs) (Hobo et al., 1999) were identified in 6 Shaker K^+^ channel genes. The *SiKAT1* had 9 ABRE elements, while *SiAKT2/3* had 8. The Methyl jasmonate (MJ)-responsive elements CGTCA-motif and TGACG-motif (Wang et al., 2019) were found in 9 genes. The *SiAKT1* had the largest number of these two elements (6 for each). Other phytohormone-related cis-acting elements were identified in different genes, including AuxRR-core, TGA-element (auxin-responsive element) (Ulmasov et al., 1997), TCA-element (salicylic acid (SA) responsiveness; Zhang R. X. et al., 2017), GARE-motif, P-Box (gibberellin (GA3) responsive element; Washida et al., 1999), and ERE (ethylene-responsive element; Fujimoto et al., 2000), suggesting Shaker K^+^ channel genes were under the regulation of different phytohormones. Many cis-acting elements involved in plant stress response were also found in the promotor region of Shaker K^+^ channel genes. MYB and MYC, related to drought stress and ABA regulation (Abe et al., 1997), were predicted in all genes. Moreover, the MYC element was found in the largest number in Group II. The G-Box element was involved in chlorophyll synthesis (Abe et al., 1997) and was the most abundant in *SiKAT3*. The WUN-motif, a wound-responsive element (Hayashi et al., 2003), was only found in *SiAKT1* and *SiSKOR*. In conclusion, cis-acting element analysis showed that most Shaker K^+^ channel genes responded to different phytohormones and environmental stresses.

### Prediction of Post-translational Modification Sites and Abscisic Acid Binding Sites on Shaker K^+^ Channels in Foxtail Millet

Protein phosphorylation and asparagine (*N*)-linked glycosylation are two important post-translational modifications that affect the protein stability, protein subcellular location, and protein-protein interaction in plants (Xu et al., 2019). They are crucial for plant response to environmental stresses (Jiao et al., 2020). As shown in Supplementary Figure 3, these two kinds of post-translational modifications in Shaker K^+^ channel proteins from foxtail millet were predicted. SiKAT3 and SiAKT1 contained the highest number of glycosylation sites (five sites). SiKC1a, SiKC1b, and SiSKOR have three sites. SiAKT2 and SiAKT2/3 were predicted to have two sites, and the remaining proteins contained only 1 glycosylation site. Regarding the predicted phosphorylation sites, the Shaker K^+^ channel proteins in foxtail millet ranged from 40 to 79. There were 79 sites in AKTs (SiAKT1 and SiAKT2), while 40 and 45 were predicted in SiKC1a and SiKC1b, respectively.

The plant hormone ABA plays an important role in plant response to abiotic stress, especially drought stress (Danquah et al., 2014; Fahad et al., 2015). Previous reports have shown that the GORK of Arabidopsis harbors potential ABA interaction sites (GORK^N558, K559, Y562, R565^). The mutation of K559 and Y562 to Ala significantly reduces the outward K^+^ current of GORK, while the other two mutations of N558A and R565A lead to the complete loss of GORK’s channel function (Ooi et al., 2017). The relationship between outward rectifying K^+^ channel SKOR and GORK is close in evolution. Therefore, we compared the GORK and SKOR protein sequences of foxtail millet, Arabidopsis, tobacco, and other plants (rice, sorghum, and wheat). As shown in Supplementary Figure 3C, these four amino acid sites existed on the GORK protein of most species. Compared to the sequence of GORK in Arabidopsis, only N558 changed to Ser in SiGORK, while the N558 and K559 changed to Lys-Asn in SiSKOR. Considering that Asn and Ser are polar amino acids with uncharged R groups, it is still possible that SiGORK contains a potential ABA binding site.

### *In silico* Transcript Analysis of the Shaker K^+^ Channel Genes in Foxtail Millet

**FIGURE 3:**
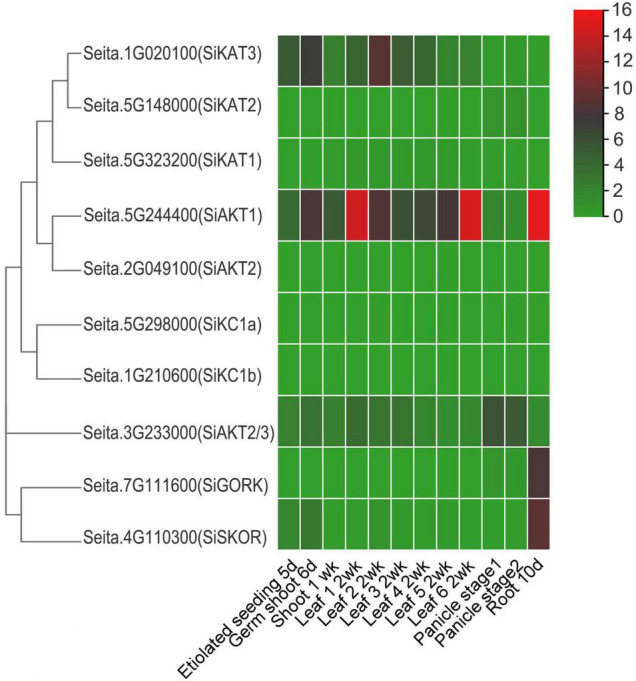
Expression profile of Shaker K^+^ channel genes in different tissues and developmental stages of foxtail millet. Gene expression data is downloaded from the GeneAtlas v1 Tissue Sample (Phytozome 13), including etiolated seeding (5 days), germ shoot (6 days), shoot (1 week), leaf (different leaf at 2 weeks), panicle stage 1 and 2, and root (10 days). The color scale represents expression levels (FPKM value) from high (red) to low (green color).

To analyze the expression pattern of Shaker K^+^ channel genes in different tissues, publicly available foxtail millet RNA-seq data (GeneAtlas v1 Tissue Sample from Phytozome 13) was used. This dataset included information from various tissues, including etiolated seeding at 5 days, germ shoot at 6 days, shoot at 1 week, different leaf (1–6) at 2 weeks, panicle stage 1 and 2, and root at 10 days. As shown in Figure 3, the *SiAKT1* was expressed highly in various tissues, especially in leaf and root, suggesting its role in K^+^ uptake and transport between tissues. The *SiKAT3* could be found in all tissues above ground. The *SiAKT2/3* was expressed highly in panicle. The two genes in group V (*SiGORK* and *SiSKOR*) were highly expressed in roots. The *SiKAT1*, *SiAKT2*, *SiKC1a*, and *SiKC1b* were almost not detected in all tissues.

### Expression Analysis of Shaker K^+^ Channels Under Different Abiotic Stresses

To further study the role of Shaker K^+^ channels in foxtail millet stress resistance, 14-days-old seedlings of “Jingu21” were subjected to different abiotic treatments (cold, heat, NaCl, and PEG). The seedlings under temperature stresses did not show any significant difference after 24 h (Supplementary Figure 4). However, after 24 h of other abiotic stresses, the leaves of seedlings turned yellow and wrinkled, especially under high levels of salt and PEG treatments. Then the leaf samples were harvested. The expression patterns of channel genes were detected by qRT-PCR. The transcription levels of four genes (*SiKAT1, SiAKT2, SiKC1a*, and *SiKC1b*) were not detectable, which was consistent with the pattern shown in Figure 3. Considering that many genes were expressed with circadian rhythms, we also tested the transcription of these channels without abiotic stresses. The results were shown in Supplementary Figure 5. There were no significant changes between 0 and 12 or 24 h in either soil culture (A) or liquid medium culture (B).

**FIGURE 4:**
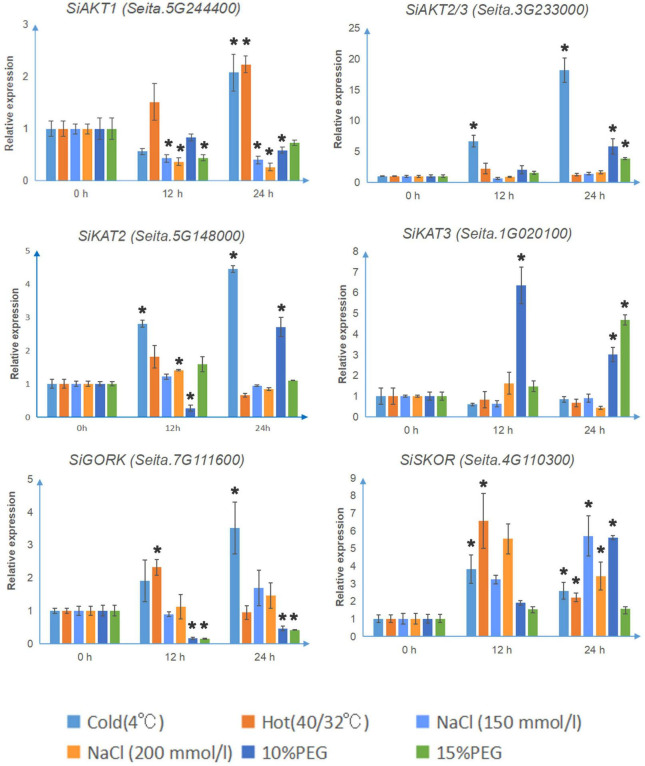
The relative transcript levels of Shaker K^+^ channel genes in foxtail millet under different abiotic stresses. The qRT-PCR analyses were used to access transcript levels of Shaker K^+^ channel genes from foxtail millet with or without 12 and 24 h cold (4°C), hot (40°C day/32°C night), salt (150 mmol/l, 200 mmol/l NaCl), and osmotic stress (10 and 15% PEG) treatments. Each bar represents the mean ± SE normalized to *SiAct2 (Seita.8G043100)*. All samples were run in three biological and three technical replicates. Asterisk indicates that the gene expression under stress has a significant difference compared with the control at 0 h (*p < 0.05).

As shown in Figure 4, under cold stress (4°C), the transcription levels of *SiAKT1, SiAKT2/3*, *SiKAT2, SiSKOR*, and *SiGORK* increased significantly after 24 h treatment. The *SiSKOR*’s transcription level was up-regulated more after 12 h cold treatment than after 24 h. Under hot stress (40°C day/32°C night), *SiAKT1* was up-regulated after 24 h treatment. The transcription levels of *SiSKOR* and *SiGORK* increased strongly after 12 h treatment. Under salt stress (150 mmol/l, 200 mmol/l NaCl) or PEG (10 and 15%) treatments, transcription of Shaker K^+^ channel genes in foxtail millet varied. The transcription level of *SiAKT1* decreased under both treatments. *SiAKT2/3* was up-regulated after 24 h treatment of PEG. For *SiKAT2*, 200 mmol/l salt stress induced its transcription at 12 h; 10% PEG treatment decreased its expression at 12 h, but significantly up-regulated it after 24 h. *SiKAT3* was strongly induced by 10% PEG treatment at 12 h. At 24 h, this gene was induced by both 10 and 15% PEG treatments. *SiGORK* decreased under PEG treatment in both time points, while *SiSKOR* was up-regulated by salt stress and 10% PEG treatments after 24 h.

### Expression Analysis of Shaker K^+^ Channels Under Different Phytohormones

Considering that the promoters of Shaker K^+^ channel genes in foxtail millet contain many phytohormone-related elements (As shown in Supplementary Figure 2), the transcription patterns of channel genes under different phytohormone treatments were analyzed. After 24 h of phytohormone treatments, the “Jingu21” seedlings did not show any significant difference (Supplementary Figure 4). Again, four genes (*SiKAT1, SiAKT2, SiKC1a*, and *SiKC1b*) were not detected under treatments.

**FIGURE 5:**
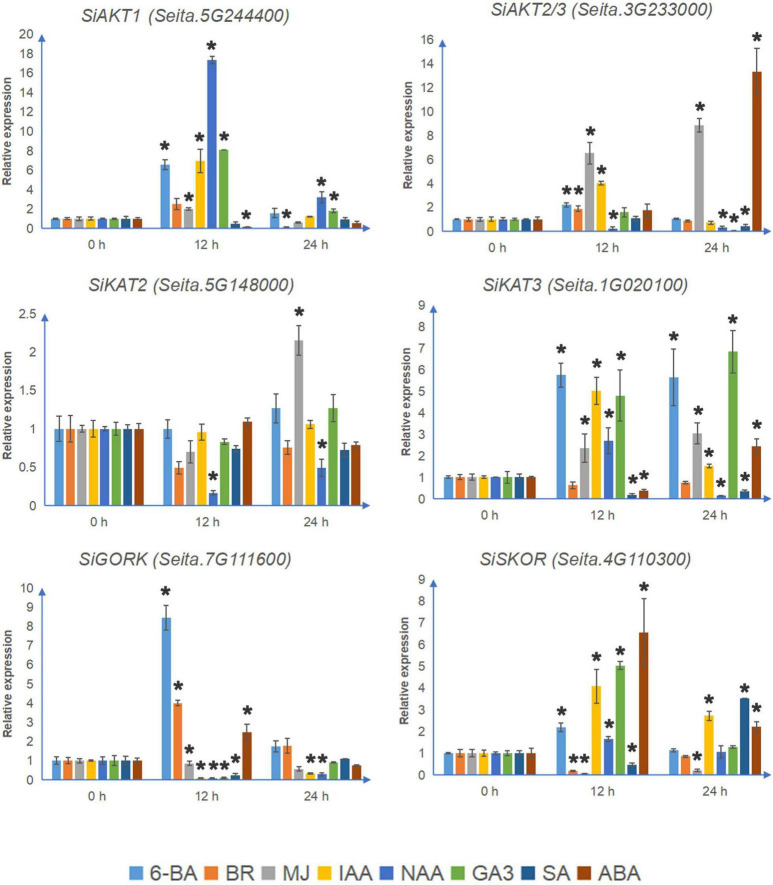
The relative transcript levels of Shaker K^+^ channel genes in foxtail millet under different phytohormone treatments. The qRT-PCR analyses were used to access transcript levels of Shaker K^+^ channel genes from foxtail millet with or without 12 and 24 h ABA (100 μmol/l), 6-BA (75 μmol/l), IAA (10 μmol/l), NAA (10 nmol/l), BR (100 μmol/l), GA3 (1 mmol/l), MJ (100 μmol/l), and SA (10 mmol/l) treatments. Each bar represents the mean ± SE normalized to *SiAct2 (Seita.8G043100)*. All samples were run in three biological and three technical replicates. Asterisk indicates that the gene expression under stress has a significant difference compared with the control at 0 h (*p < 0.05).

Most Shaker K^+^ channel genes contain the MeJA-responsive elements CGTCA-motif and TGACG-motif (Supplementary Figure 2). As shown in Figure 5, under MJ treatment, the transcription levels of all 4 detectable inward K^+^ channel genes were up-regulated. MJ suppressed both *SiGORK* and *SiSKOR*. Auxin treatment induced expression of *SiAKT1*, *SiKAT3*, and *SiSKOR*, but decreased the level of *SiKAT2* and *SiGORK*. Gibberellin treatment also increased the transcription of *SiAKT1, SiKAT3*, and *SiSKOR*, but only inhibited the expression of *SiAKT2/3*. In the case of salicylic acid treatment, the transcription of *SiAKT2/3* and *SiKAT3* were down-regulated. *SiSKOR* was significantly inhibited at 12 h but induced at 24 h under SA treatment. Six channel genes contained the ABRE element. Among them, *SiAKT2/3* and *SiKAT3* were induced by ABA treatment, while the level of *SiAKT1* was suppressed at 12 h. We also tested the response of channel genes to 6-BA and BR. The former induced transcription of all six detectable channels except *SiKAT2*. The latter up-regulated *SiAKT2/3* and *SiGORK* but suppressed *SiSKOR* and *SiAKT1* at 12 h.

**FIGURE 6:**
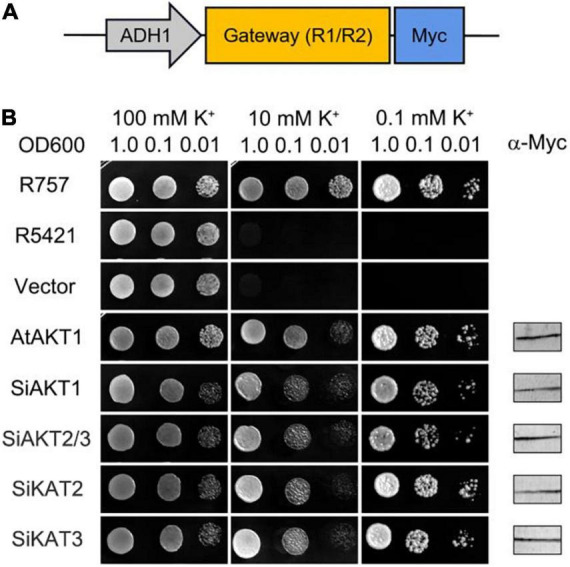
Shaker K^+^ channels from foxtail millet mediate K^+^ Transport in Yeast. **(A)** Schematic of the padh1-Dest vector. **(B)** AtAKT1 and Shaker K^+^ channels from foxtail millet complement the K^+^ uptake-deficient yeast mutant R5421. The yeast strain R757 was used as a control. Immunoblot analysis (5 μg total protein/lane) of yeast was performed using a commercial α-Myc antibody.

Under different abiotic stresses or phytohormone treatments, the transcription of *SiKC1s* was still missing. Whether this is a phenomenon that only existed in “Jingu21?” We performed the RT-PCR analysis in the other two foxtail millet cultivars “Longgu16” and “Jigu39,” to answer this question. One of the results was shown in Supplementary Figure 6. In either roots or leaves of different cultivars, we could not detect the expression of *SiKC1s*.

Taken together, *SiAKT1, SiKAT3, SiGORK*, and *SiSKOR* were more worth further research due to their significant changes under most stresses and phytohormone treatments.

### K^+^ Transport Activity of Shaker K^+^ Channels in Foxtail Millet

The inward K^+^ transport activities of four detectable Shaker K^+^ channels in foxtail millet were tested in the K^+^-deficient yeast mutant strain R5421 (*trk1Δ, trk2Δ*), in which two K^+^ transporter genes (TRK1 and TRK2) were deleted (Gaber et al., 1988; Li et al., 2014). Shaker K^+^ channel genes were inserted into the padh1-Dest vector and transformed into R5421. *AtAKT1 (At2g26650)* from Arabidopsis was used as the positive control. The wild-type yeast strain R757 was used as the growth control. The yeast growth assays were performed on a CSM medium with different concentrations of K^+^. Figure 6 showed one of three independent experiments which yielded similar results. Under high K^+^ concentration (100 mmol/l), all yeast grew well. Under low K^+^ concentration (0.1 mmol/l), there was no growth recovery of R5421 and R5421 containing the empty vector. The K^+^ channels from foxtail millet or Arabidopsis rescued R5421 yeast growth, suggesting that the Shaker K^+^ channels we tested had similar K^+^ uptake function as the AKT1 channel in Arabidopsis.

### The Interaction Between SiSNARE Proteins and Shaker K^+^ Channel SiKAT2 in Foxtail Millet

**FIGURE 7:**
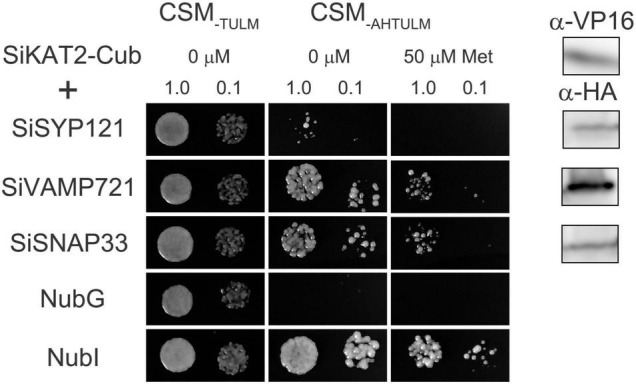
SiKAT2 selective interact with SiSNARE proteins. Yeast mating-based split-ubiquitin assay for the interaction of SiSYP121, SiVAMP721, and SiSNAP33 with SiKAT2-Cub as baits. After several attempts, SiKAT3 failed to transform into yeast. Yeast diploids were created with NubG fusion constructs of each of the SiSNARE proteins together with controls (NubG, negative; NubI, positive) spotted (left to right) on CSM medium without Leu, Trp, Ura, and Met (CSM_–TULM_) to verify mating, CSM medium without Leu, Trp, Ura, Met, Ade, and His (CSM_–AHTULM_) to verify adenine- and His-independent growth, and on CSM_–LTUMAH_ with the addition of Met to verify interaction at lower K^+^ channel-Cub expression levels. Diploid yeast was dropped at 1.0 and 0.1 OD600 in each case. Immunoblot analysis (5 μg total protein/lane) of the haploid yeast used for mating (right) using commercial α-HA antibody for the SiSNARE fusions and α-VP16 antibody for the K^+^ channel fusions.

Previous reports have shown that ion channels are regulated by SNARE proteins (Karnik et al., 2017; Zhang et al., 2020). In Arabidopsis, SYP121 and VAMP721 form the SNARE complex during membrane fusion. They interact with KC1 and KAT1 K^+^ channels and regulate the channel activity in an opposite way (Honsbein et al., 2009; Zhang et al., 2015). SNAP33 also interacts with K^+^ channels and regulates channel activity with SYP121 and VAMP721 (Waghmare et al., 2019). We analyzed foxtail millet SNARE-Shaker K^+^ channel interaction by mbSUS assay. *SiKAT1* was almost undetected in all tissues (Figure 3). Thus, it could not be cloned. *SiKAT3* failed to transform into yeast. In this assay, Shaker K^+^ channel SiKAT2 was fused with C-terminal halves of the ubiquitin (Cub) and used as bait, while SiSYP121, SiVAMP721, and SiSNAP33 were fused with N-terminal halves of the ubiquitin (Nub) and used as prey. The protein-protein interaction will reassemble ubiquitin, release trans-activator, and activate reporter genes, allowing yeast growth on selective media. This method has been previously used in membrane protein interaction analysis (Honsbein et al., 2009; Grefen et al., 2010; Zhang et al., 2015, 2019; Zhang B. et al., 2017). Figure 7 showed that diploid yeast expressing SiKAT2 with SiVAMP721 or SiSNAP33 grew well on selective media, even on the media containing 50 μmol/l Met (which suppresses bait protein expression). However, diploid yeast growth was not recovered with SiSYP121 as prey. This result demonstrated the selective interaction between the Shaker K^+^ channel and SNARE proteins in foxtail millet.

## Discussion

Potassium (K^+^) is involved in stress resistance in higher plants (Wang et al., 2013). Shaker K^+^ channels are highly conserved voltage-dependent ion channels in plants and play an important role in K^+^ absorption and transport. Investigations focused on this family of genes have been performed in many species (Bauer et al., 2000; Amrutha et al., 2007; Dreyer and Uozumi, 2011; Jin et al., 2021). The current study identified 10 Shaker K^+^ channel family members in foxtail millet. In 14-days-old seedlings of “Jingu21,” six of 10 Shaker K^+^ channels were detectable. Their inward K^+^ transport activities were verified in yeast. The qRT-PCR analysis showed that the transcriptions of channels responded to different abiotic stresses and phytohormone treatments. Compared to their homologous in Arabidopsis, there were different selective interactions between SNARE proteins and Shaker K^+^ channel SiKAT2 in foxtail millet.

Previous research suggested that Shaker K^+^ channels are highly conserved in plants (Dreyer and Blatt, 2009). In the present study, Shaker K^+^ channel genes from foxtail millet were classified into five groups as reported in other species (Table 1 and Figure 1). Further analysis showed that channel genes of each group shared similar motifs and gene structures (Figure 2). Based on the phylogenetic analysis, the channel genes from foxtail millet were more similar to their orthologous genes in rice than that in Arabidopsis, suggesting the diversification of Shaker K^+^ channels happened before the separation of monocots and dicots in evolution and was consistent with the previous report (Pilot et al., 2003b). The five-group classification of channels is based on their functional diversity (Pilot et al., 2003b; Dreyer and Blatt, 2009). We predicted each group’s K^+^ transport activity in foxtail millet based on their classification.

To identify the roles of Shaker K^+^ channels in abiotic stress response, we first analyzed the cis-acting elements in their promoter region. As shown in Supplementary Figure 2, most of the genes contained MYB and MYC elements, suggesting that the Shaker K^+^ channels in foxtail millet were regulated by drought stress. We also found various hormone response elements, including ABA, auxin, GA3, MeJA, and ethylene. Various cis-acting elements existed in Shaker K^+^ channel genes, even in the same group, suggesting the diversity of these genes’ expression in response to environmental stresses.

Expression profiling of Shaker K^+^ channels was also performed using previously released RNA-seq data (Figure 3). Shaker K^+^ channel genes showed various expression levels in different tissues. *SiAKT1* was expressed in all tissues, especially in roots, supporting its role in K^+^ absorbing from the soil as its orthologous genes in other species (Véry and Sentenac, 2003; Li et al., 2014). In Arabidopsis, KC1 in group IV is silent when expressed alone but function as a general regulator of channel activity when it forms hetero-tetramer with other channel proteins (Duby et al., 2008; Jeanguenin et al., 2011). Interestingly, according to the RNA-seq data, the *SiKC1s* could not be detected in all tissues. One explanation of this result is that the expression of some K^+^ channels is only induced under stress (Zhang et al., 2018; Jin et al., 2021). We will go back to this point later.

Glycosylation and phosphorylation modifications play vital roles in protein functions. The potential post-translational modifications of Shaker K^+^ channel proteins in foxtail millet were also predicted. Our results showed that SiAKT1 and SiAKT2 had the largest number of modification sites (Supplementary Figure 3). Previous reports have shown that AtAKT1 was activated by phosphorylation, which depends on the calcineurin B-like 1 (CBL1) with the CBL-interacting protein kinase (CIPK 23) (Xu et al., 2006; Honsbein et al., 2009). Such phosphorylation activating might also happen in SiAKTs and other Shaker K^+^ channels in foxtail millet.

As mentioned above, to investigate whether Shaker K^+^ channels in foxtail millet, especially SiKC1s, were regulated by abiotic stresses or phytohormone treatments, we performed the qRT-PCR analysis in 14-days-old seedlings of “Jingu21” under different treatments (cold, heat, NaCl, PEG, ABA, 6-BA, IAA, NAA, BR, GA3, MJ, and SA). “Jingu21” was one of the cultivars produced by radiation-induced mutagenesis in breeding programs (He et al., 2015). This cultivar shows good quality, high yield, and drought stress-tolerant than the other cultivars (Wang et al., 2021). Our results showed that four Shaker K^+^ channel genes (*SiKAT1, SiAKT2, SiKC1a*, and *SiKC1b*) were not detected in “Jingu21” under different treatments (Figures 4, 5). This finding supports the RNA-seq data shown in Figure 3. In Arabidopsis, KC1 forms the functional channel with inward Shaker K^+^ channel subunit AKT1. It performs as an important regulator of inward K^+^ current by negatively shifting the channel activation threshold, which helps to limit the K^+^ leakage under low K^+^ condition (Honsbein et al., 2009; Li et al., 2014). To investigate whether the missing *SiKC1s*’ transcription was related to the cultivar, we performed the RT-PCR analysis in the other two foxtail millet cultivars, “Longgu16” and “Jigu39.” Our previous research has shown that “Longgu16” was more sensitive to drought stress than “Jingu21” (Wang et al., 2021). The results here showed that the transcription of *SiKC1s* could not be detected in different cultivars of foxtail millet (Supplementary Figure 6). The undetectable *SiKC1s* left a question mark in the present study. *KC1* was up-regulated by salt stress and K^+^ deficiency in Arabidopsis (Pilot et al., 2003a). The transcription level of *SiKC1s* under low K^+^ stress was worth testing in future research. SiAKT1 might form a functional channel with other Shaker K^+^ channel proteins in foxtail millet was another explanation for the missing *SiKC1s* here. *SiAKT2/3* is found in most tissues (Figure 3). It is worth co-expressing *SiAKT2/3* with *SiAKT1* and detecting the channel gating by the electrophysiological experiment. In rice, OsAKT1 is not regulated by the KC1-like gene (Fuchs et al., 2005; Li et al., 2014). Thus, Li et al. found that OsAKT1 alone could restrain the K^+^ leakage under low K^+^ (Li et al., 2014). OsAKT1 is affected by some other mechanisms (like phosphorylation and vitamin B6), which might help to modulate K^+^ uptake together (Lee et al., 2007; Xia et al., 2014). It is worth further investigating whether similar mechanisms happened in foxtail millet.

As shown in Figure 4, the other remaining genes displayed their regulation pattern under different treatments, consistent with findings from previous reports on Arabidopsis (Pilot et al., 2003a) and sweet potato (Jin et al., 2021). In Arabidopsis, AKT1 is involved in a major pathway for K^+^ uptake at the root epidermis (Geiger et al., 2009). *SiAKT1* induced by temperature stress and decreased under salt and osmotic stress revealed the role of K^+^ absorption in plant adaptation to environmental conditions. GORK plays a central role in K^+^ loss from the cytosol, and the cytosol K^+^ change is an important second messenger in plant stress response (Adem et al., 2020). In foxtail millet, we found *SiGORK* was induced by cold stress but inhibited by osmotic stress, suggesting GORK is the key to understanding the foxtail millet response mechanism to these stresses.

As shown in Figure 5, Shaker K^+^ channel genes in foxtail millet responded variant to different phytohormone treatments, supported by previous reports in other species (Philippar et al., 1999; Pilot et al., 2003a; Jin et al., 2021). ZMK1 in maize is the homology to AKT1 in Arabidopsis. This channel gene was induced by auxin (NAA) treatments and involved in coleoptile elongation (Philippar et al., 1999, 2006). In the present study, we found that NAA significantly increased the transcription of *SiAKT1*. Considering the wide expression of *SiAKT1* in various tissues (Figure 3), this gene might play an important role in auxin-related plant growth. ABA serves as an important endogenous messenger in abiotic stress responses in plant. *SKOR* is regulated by different abiotic stress and decreased under ABA treatment in Arabidopsis and sweet potato (Pilot et al., 2003a; Jin et al., 2021). This channel is involved in the K^+^ secretion into the xylem sap (Johansson et al., 2006; Dreyer and Blatt, 2009). Our analysis revealed that *SiSKOR* was up-regulated by all stresses except ABA treatment. The divergent pattern of *SiSKOR* between foxtail millet and other species suggests the role of K^+^ redistribution among tissues in foxtail millet stress-tolerant. Previous reports have also shown that ABA does not affect the transcription level of *GORK* but induces plasma membrane depolarization and activates GORK in guard cells, leading to stomatal closure (Thiel et al., 1992; Hosy et al., 2003; Pilot et al., 2003a). In our result, *SiGORK* was only induced by ABA at 12 h. In 2017, Ooi et al. has reported that direct GORK-ABA interaction enhances K^+^-efflux current through GORK (Ooi et al., 2017). So, we performed a protein sequence alignment between GORK and SKOR from different species. We found that the important sites for ABA docking existed in most species, especially foxtail millet (Supplementary Figure 3), suggesting these outward channels in foxtail millet might also be under the regulation of ABA through directly binding.

As shown in Figure 6, the yeast complementation helped to confirm that the detectable inward K^+^ channels had K^+^ transport activity. This result supported the five-group classification of Shaker K^+^ channels in foxtail millet.

Previous reports have shown that KAT1 interacts with SNARE proteins, including SYP121, VAMP721, and SNAP33 in Arabidopsis, and these SNARE proteins co-modulate the channel activities (Honsbein et al., 2009; Karnik et al., 2017; Waghmare et al., 2019). We found that SiKAT2 interacted with SiVAMP721 and SiSNAP33, suggesting similar SNARE-K^+^ channel interactions in different species. However, we did not find the interaction of SiKAT2 with SiSYP121 (Figure 7). In Arabidopsis, when VAMP721 negatively regulates the channel activity, the binding of SYP121 with K^+^ channel promotes channel activity, such SYP121-VAMP721 balance is essential for K^+^ uptake and stomata movement (Honsbein et al., 2009, 2011; Zhang et al., 2015). SiSYP121 is close in evolution to SYP121 of Arabidopsis, and their sequences are highly similar (Wang et al., 2021). Surprisingly, there was no interaction between SiSYP121 and SiKAT2. There might be other SiSNARE proteins that take the role of positive K^+^ channel regulation. Identifying the difference between foxtail millet and Arabidopsis for SNARE-related K^+^ channel regulation will help to reveal the mechanism of K^+^ uptake and stress tolerance of foxtail millet.

SiKAT3 was expressed in R5421 yeast used in the complementation assay, but it failed to be expressed in THY.AP4 yeast, which was used in the mbSUS assay. We speculate that the codon bias might affect the expression of SiKAT3 in THY.AP4. There were differences between R5421 and THY.AP4 in genotype (Grefen et al., 2009; Li et al., 2014). Codon bias affects proteins’ expression level, localization, time rhythm, and function (Zhou et al., 2013; Engel et al., 2021). Grefen et al. have optimized the codons of AKT1 of Arabidopsis and transferred it into THY.AP4 yeast to get better protein expression (Grefen and Blatt, 2012).

In conclusion, we identified ten Shaker K^+^ channel genes in foxtail millet and analyzed their roles in plant stress responses. This work will facilitate further research focused on the biological roles of Shaker K^+^ genes in foxtail millet, which is beneficial to realize the combination of theoretical research in model plants and application research in crops.

## Data Availability Statement

The original contributions presented in this study are included in the article/Supplementary Material, further inquiries can be directed to the corresponding author/s.

## Author Contributions

BZ, LZ, PY, and YG designed the experiments. YG, HW, XW, and ML performed the experiments. BZ, HW, YG, and LZ analyzed the data and wrote the manuscript. All authors read and approved the final manuscript.

## Conflict of Interest

The authors declare that the research was conducted in the absence of any commercial or financial relationships that could be construed as a potential conflict of interest.

## Publisher’s Note

All claims expressed in this article are solely those of the authors and do not necessarily represent those of their affiliated organizations, or those of the publisher, the editors and the reviewers. Any product that may be evaluated in this article, or claim that may be made by its manufacturer, is not guaranteed or endorsed by the publisher.

## Footnotes

1
http://www.arabidopsis.org/

2
http://rice.plantbiology.msu.edu/index.shtml

3
http://pfam.xfam.org/

4
http://smart.embl-heidelberg.de/

5
http://web.expasy.org/protparam

6
https://wolfpsort.hgc.jp/

7
http://www.cbs.dtu.dk/services/TMHMM

8
http://meme.nbcr.net/meme/

9
http://gsds.cbi.pku.edu.cn

10
http://bioinformatics.psb.ugent.be/webtools/plantcare/html

11
http://www.cbs.dtu.dk/services/NetOGlyc/

12
http://www.cbs.dtu.dk/services/NetPhos/

## Supplementary Material

Supplementary Figure 1 Chromosomal localization of Shaker K^+^ channel genes from foxtail millet. The location information of 10 Shaker K^+^ channel genes on the chromosome was obtained from the Phytozome database (*Setaria italica* v2.2). Different colors on the nine chromosomes (chr1–chr9) indicate different channel groups.

Supplementary Figure 2 Cis-acting element analysis of the promoter regions of Shaker K^+^ channel genes from foxtail millet. The number of each cis-acting element in the promoter regions (2,000 bp upstream of the translation start site) of Shaker K^+^ channel genes was shown. Based on the functional annotations, the cis-acting elements were classified into three major classes: plant growth and development, phytohormone responsiveness, and abiotic/biotic stresses related cis-acting elements.

Supplementary Figure 3 Prediction of post-translational modification sites and ABA binding sites in the amino acid sequences of Shaker K^+^ channel proteins from foxtail millet. **(A)** *N*-glycosylation site; **(B)** phosphorylation site; **(C)** ABA binding sites in GORK and SKOR.

Supplementary Figure 4 The phenotype of “Jingu21” seedlings after 24 h treatment of different abiotic stresses **(A)** or phytohormones **(B)**.

Supplementary Figure 5 The transcript levels of six Shaker K^+^ channels did not change without abiotic stresses. **(A)** Seedlings of “Jingu 21” grow in soil (control for cold or hot treatments). **(B)** Seedlings of “Jingu 21” grow in liquid medium (control for salt, PEG, and phytohormone treatments). The Student’s *t*-test was used to analyze statistical significance. Compared to that at 0 h, the transcript levels of Shaker K^+^ channels at 12 and 24 h showed no difference (*P* > 0.05).

Supplementary Figure 6 The transcripts of *SiKC1a* and *SiKC1b* could not be detected by RT-PCR in different cultivates. The transcripts of *SiKC1a (Seita.5G298000)* and *SiKC1b (Seita.1G210600)* were tested by RT-PCR in 14 days old seedlings of “Jingu21,” “Longgu16,” and “Jigu39.” R means root, and L means leaf. *SiAKT1* was used as positive control. *SiAct2* was used as the internal reference. In all three cultivates, there are similar strong bands for *SiAKT1*, supporting the RNA-seq analysis in Figure 3.
