# Supplementary figures and images for: Identification and Characterization of Shaker K+ Channel Gene Family in Foxtail Millet (Setaria italica) and Their Role in Stress Response
Source: Front Plant Sci. 2022 Jun 9;13:907635. doi: 10.3389/fpls.2022.907635 (PMC9218596; doi:10.3389/fpls.2022.907635)

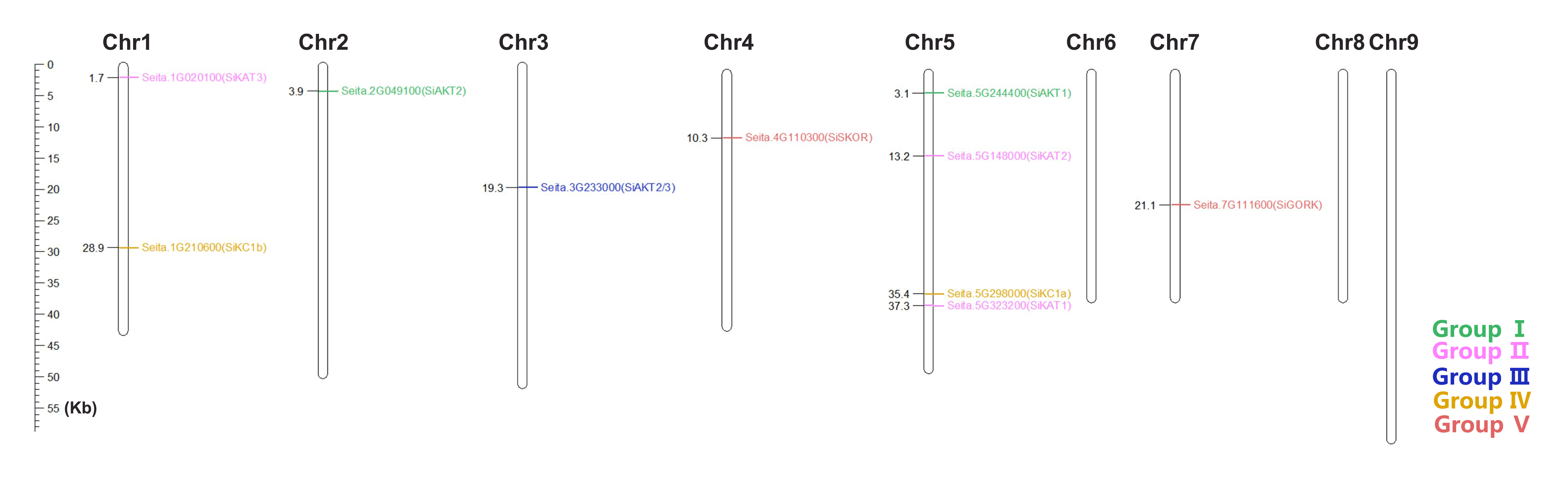

Supplement: Supplementary Figure 1 — Chromosomal localization of Shaker K+ channel genes from foxtail millet. The location information of 10 Shaker K+ channel genes on the chromosome was obtained from the Phytozome database (Setaria italica v2.2). Different colors on the nine chromosomes (chr1–chr9) indicate different channel groups. [file Image_1.JPEG]

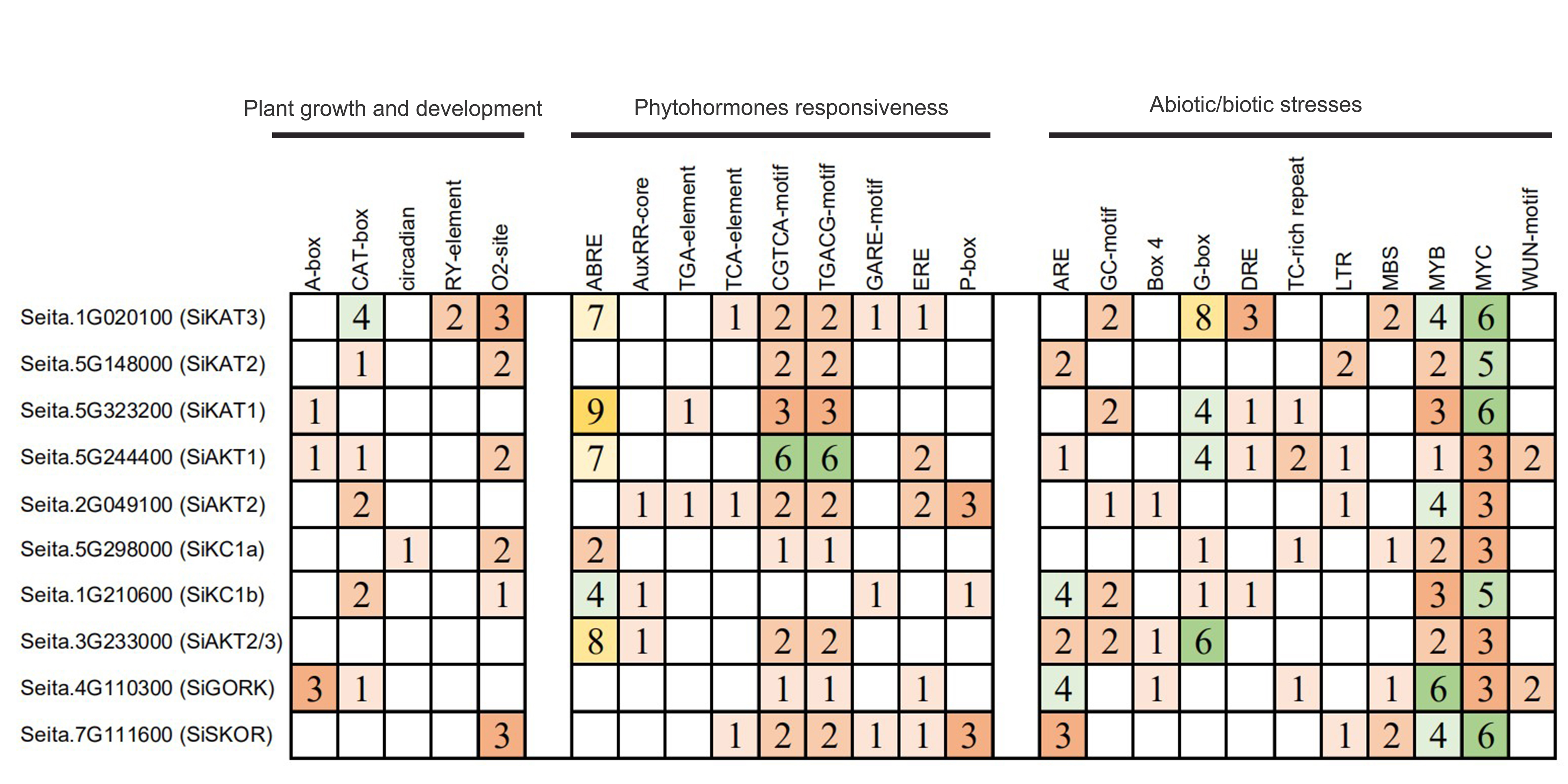

Supplement: Supplementary Figure 2 — Cis-acting element analysis of the promoter regions of Shaker K+ channel genes from foxtail millet. The number of each cis-acting element in the promoter regions (2,000 bp upstream of the translation start site) of Shaker K+ channel genes was shown. Based on the functional annotations, the cis-acting elements were classified into three major classes: plant growth and development, phytohormone responsiveness, and abiotic/biotic stresses related cis-acting elements. [file Image_2.JPEG]

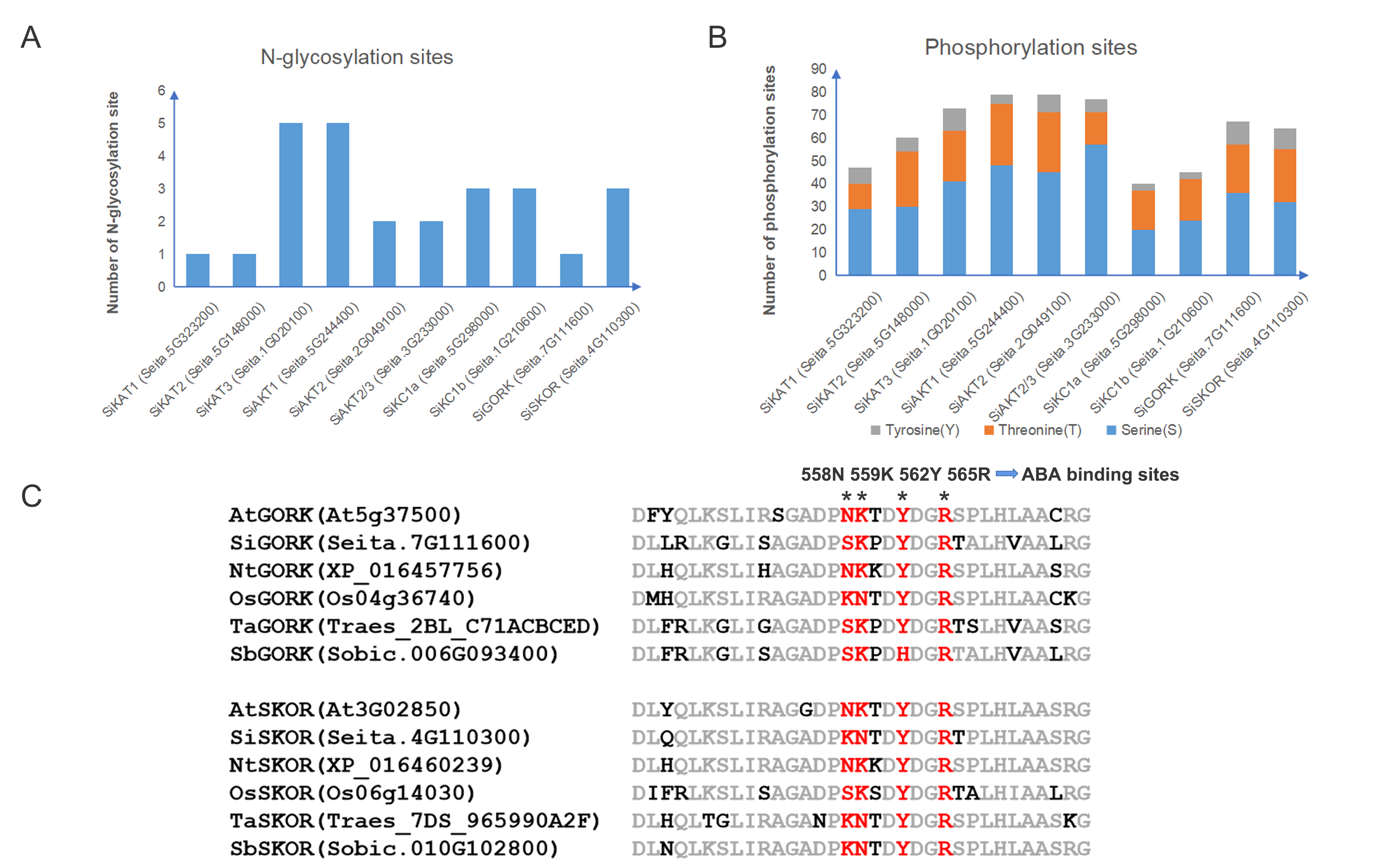

Supplement: Supplementary Figure 3 — Prediction of post-translational modification sites and ABA binding sites in the amino acid sequences of Shaker K+ channel proteins from foxtail millet. (A) N-glycosylation site; (B) phosphorylation site; (C) ABA binding sites in GORK and SKOR. [file Image_3.JPEG]

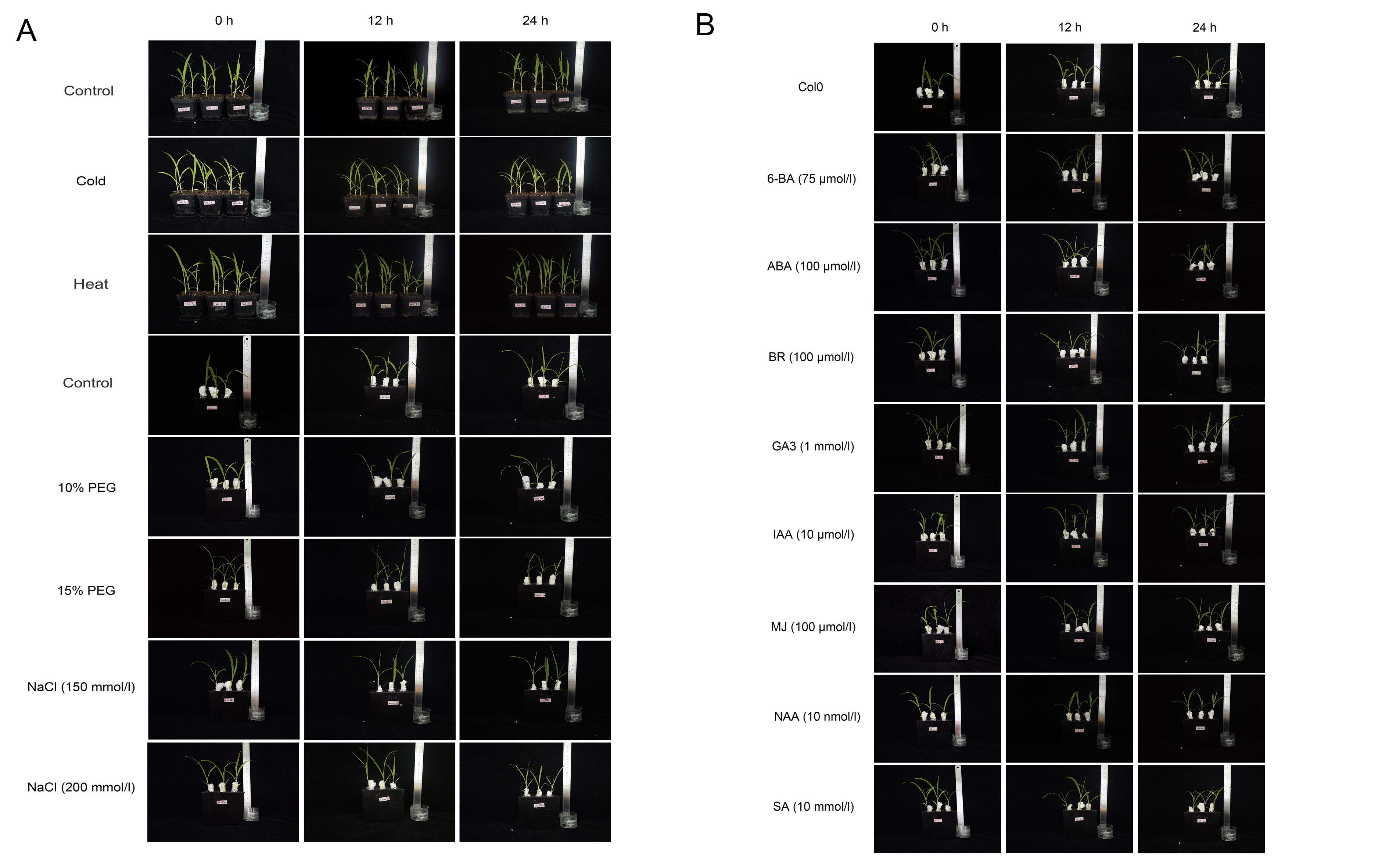

Supplement: Supplementary Figure 4 — The phenotype of “Jingu21” seedlings after 24 h treatment of different abiotic stresses (A) or phytohormones (B). [file Image_4.JPEG]

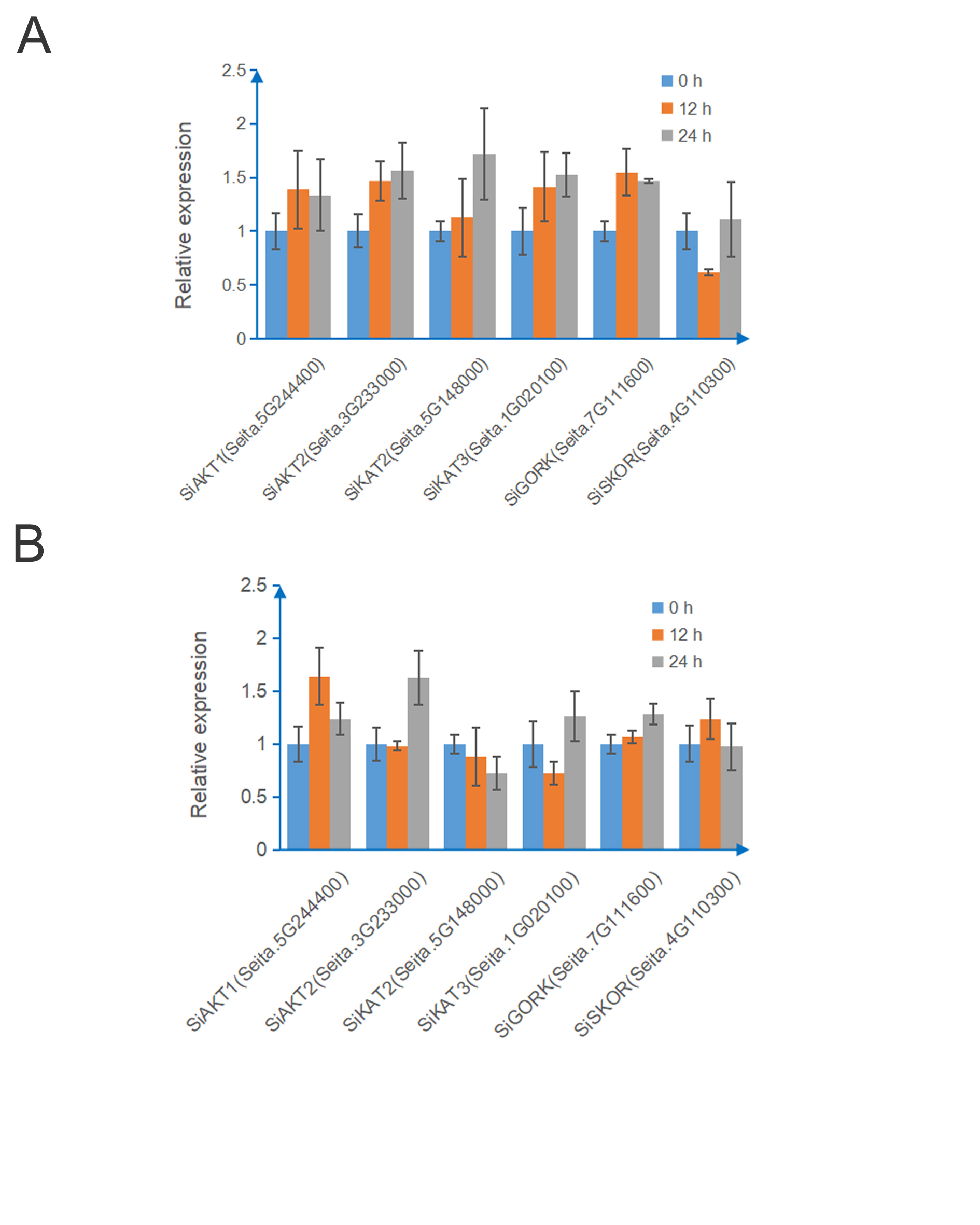

Supplement: Supplementary Figure 5 — The transcript levels of six Shaker K+ channels did not change without abiotic stresses. (A) Seedlings of “Jingu 21” grow in soil (control for cold or hot treatments). (B) Seedlings of “Jingu 21” grow in liquid medium (control for salt, PEG, and phytohormone treatments). The Student’s t-test was used to analyze statistical significance. Compared to that at 0 h, the transcript levels of Shaker K+ channels at 12 and 24 h showed no difference (P > 0.05). [file Image_5.JPEG]

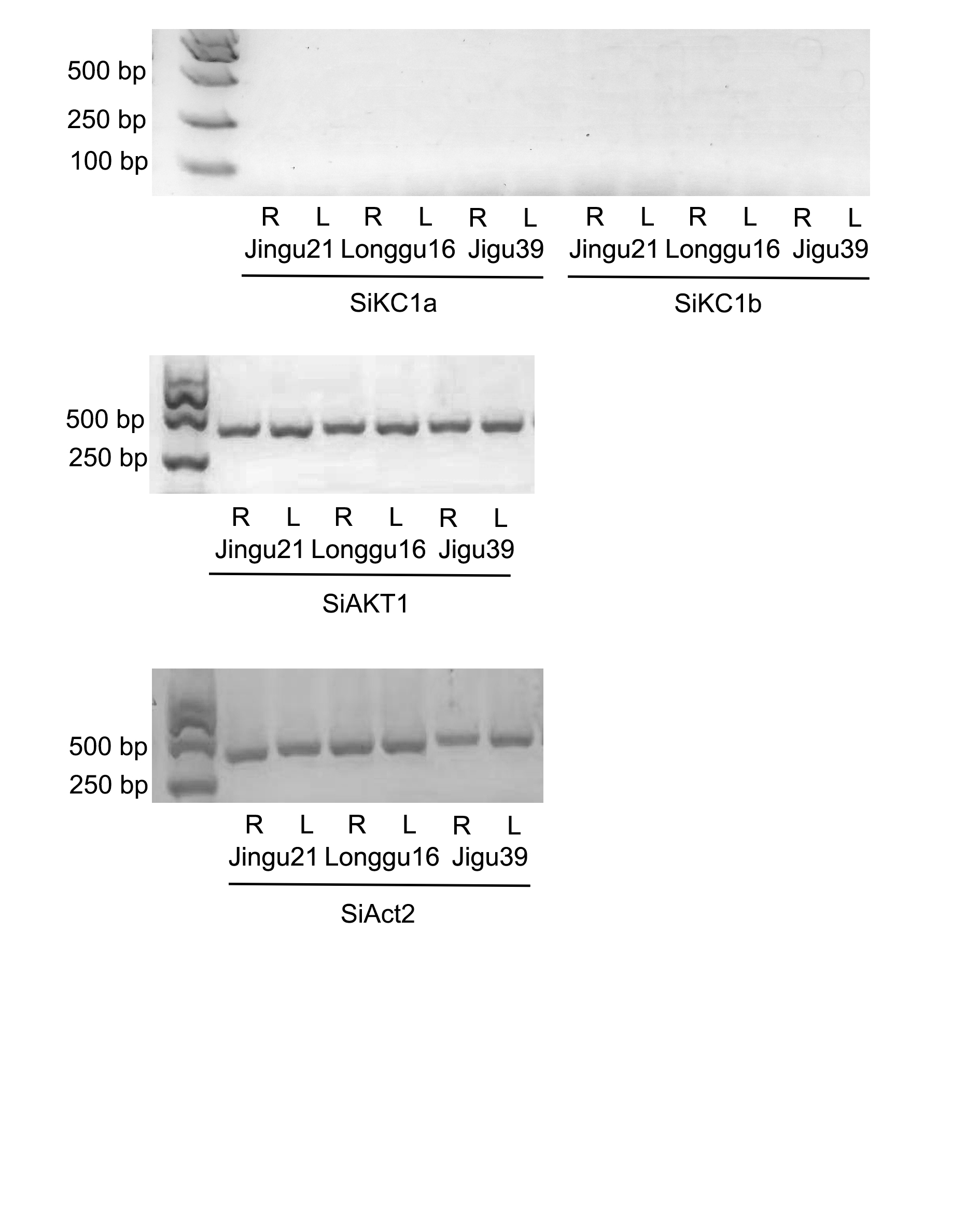

Supplement: Supplementary Figure 6 — The transcripts of SiKC1a and SiKC1b could not be detected by RT-PCR in different cultivates. The transcripts of SiKC1a (Seita.5G298000) and SiKC1b (Seita.1G210600) were tested by RT-PCR in 14 days old seedlings of “Jingu21,” “Longgu16,” and “Jigu39.” R means root, and L means leaf. SiAKT1 was used as positive control. SiAct2 was used as the internal reference. In all three cultivates, there are similar strong bands for SiAKT1, supporting the RNA-seq analysis in Figure 3. [file Image_6.JPEG]
